# Feasibility of portal or superior mesenteric vein resection and reconstruction by allogeneic vein for pancreatic head cancer—a case-control study

**DOI:** 10.1186/s12876-018-0778-y

**Published:** 2018-04-16

**Authors:** Xing-mao Zhang, Jie Zhang, Hua Fan, Qiang He, Ren Lang

**Affiliations:** 1 Department of hepatobiliary surgery, Beijing Chaoyang Hospital, Capital Medical University, 8 Gongti South Street, Chaoyang District, Beijing, 100021 China; 2 The First Hospital of Combination of the Western Medicine and Traditional Chinese Medicine, Xiaozhuang Hospital, Capital Medical University, 13 Jintai Street, Chaoyang District, Beijing, 100021 China

**Keywords:** Pancreatic cancer, Vascular resection, Reconstruction, Outcome

## Abstract

**Background:**

There are few reports about resection of portal vein (PV)/superior mesenteric vein (SMV) and reconstruction by using allogeneic vein. This case-control study was designed to explore the feasibility and safety of this operation type in patients with T3 stage pancreatic head cancer.

**Methods:**

A total of 42 patients (Group A) underwent PV/SMV resection and reconstruction by using allogeneic vein were 1:1 matched to 42 controls (Group B) with other types of resection and reconstruction. The two groups were well matched.

**Results:**

There was no significantly prolonged total operation time (Group A vs. Group B [490.0 min vs. 470 min], *P* = 0.067) and increased intraoperative blood loss (Group A vs. Group B [650.0 min vs. 450 min], *P* = 0.108) was found between the two groups. R1 rate of PV/SMV was slightly reduced in group A compared to group B (4.8% vs. 14.3%, *P* = 0.137), although no significant difference was found. The incidences of main postoperative complications between the two groups were similar. A slightly increased 1-year and 2-year overall survival rate (OS) (Group A vs. Group B [1-year OS: 62.9% vs. 57.0%; 2-year OS: 31.5% vs. 25.6%], *P* = 0.501) and disease-free survival rate (DFS) (Group A vs. Group B [1-year DFS: 43.9% vs. 36.6%; 2-year DFS: 10.5% vs. 7.4%], *P* = 0.502) could be found in group A compared to group B, although the differences were not significant.

**Conclusions:**

The operation types of PV/SMV resection and reconstruction by using allogeneic vein is safety and feasible, it might have a potential benefit for patients.

## Background

With increasing incidence and mortality, cancer is the one of leading causes of death worldwide and it has become a major public problem. Among the malignant solid tumors, pancreatic cancer is characterized by low curative rate and high mortality [1–5]. Cancer Statistics in China which is completed by National Cancer Center of China shows that the estimated new cancer cases and deaths of pancreas are 90.1 and 79.4 thousand in 2015 [6]. Despite decades of effort, pancreatic cancer remains one of the most aggressive and lethal malignancies, and the five-year survival rate remains at only ~ 5% [7]. Due to biological characteristics of pancreatic cancer, also due to anatomical location of pancreas and lack of early detection tests, most of patients with localized disease have no recognizable symptoms or signs. As a result, only 15 to 25% of patients with pancreatic cancer is eligible for curative resection. Of the unresectable patients, approximately two thirds present with distant metastases and the rest presents with locally advanced disease which has surrounding vascular invasion. According to the TNM staging system of pancreatic cancer, tumor extending to portal vein (PV) or superior mesenteric vein (SMV) without involvement of celiac axis or superior mesenteric artery is defined as T3 stage. Based on performance status and preference of patients, and decision of Multi-Disciplinary Team, different therapeutic modalities including upfront surgery, neoadjuvant chemotherapy followed by surgery, chemotherapy alone, chemoradiotherapy, etc., can be selected for T3 stage pancreatic cancer which is clinically diagnosed. Of these modalities, upfront surgery is most frequently used.

Radical pancreatic surgery accompanied with PV/SMV resection, has commonly been undertaken for pancreatic cancer with infiltration of PV/SMV since the first report of pancreatoduodenectomy (PD) with resection and reanastomosis of vein in 1951 [8, 9]. Till now, there has been no consensus yet on the survival advantage of PV/SMV resection and reconstruction for pancreatic head cancer, although similar reported short-term outcomes when compared with standard PD [10–13]. In the classification of International Study Group of Pancreatic Surgery (ISGPS), a total of four types of venous reconstruction are included [14]. With the view to improving the R0 resection rate, to reaching tension-free anastomosis, to avoiding the diameter mismatch between the PV and SMV, and also to avoiding vessel stenosis, segmental resection and reconstruction by using allogeneic vein has been preferred for selected patients since 2013 in our center.

In this study, we conducted a case-control study to explore the feasibility and safety of PV/SMV resection and reconstruction by using allogeneic vein in patients with pancreatic head adenocarcinoma.

## Methods

### Patients

From January 2012 to November 2016, patients with pancreatic head cancer who underwent surgical treatment at our institution were reviewed from our prospectively collected clinical database. Clinicopathological data of patients with duct adenocarcinoma of pancreatic head with PV/SMV infiltration by postoperative pathology were used for analysis. Patients with advanced pancreatic cancer, or with T4 stage pancreatic cancer, or without PV/SMV infiltration were excluded from this study, neither patients with cancer history nor patients with neoadjuvant therapy were enrolled in this study.

Patients underwent segmental PV/SMV resection with reconstruction using allogeneic vein were 1:1 matched with patients underwent PV/SMV resection with reconstruction by other methods (direct end-to-end anastomosis, partial venous excision with direct closure, partial venous excision using a patch or artificial vascular graft). Matching criteria included age, gender, body mass index (BMI), American Society of Anesthesiology (ASA) score, concomitant disease, blood test, surgical procedures (extended PD with extended Lymphadenectomy), postoperative pathology, and adjuvant therapy (Table 1). When investigators did the manual match, they were blinded to the surgical outcomes to reduce potential selection bias.

**Table 1:** General parameters of the two groups

Parameters	Group A (*n* = 42)	Group B (*n* = 42)	*P*-value
Gender, case (%)			0.826
Male	18 (42.9)	19 (45.2)	
Female	24 (57.1)	23 (54.8)	
Age, year, median (range)	68 (43–80)	67 (45–79)	0.952
BMI, kg/m^2^, median, (range)	21.5 (17.6–28.6)	23.1 (17.3–29.7)	0.238
ASA Score (%)			0.608
2	11 (26.2)	9 (21.4)	
3	31 (73.8)	33 (78.6)	
Concomitant disease, case (%)	32 (76.2)	33 (78.6)	0.794
Abdominal operation history, case (%)	4 (9.5)	3 (7.1)	0.693
Preoperative biliary drainage, case (%)	8 (19.0)	7 (16.7)	0.776
Hemoglobin, g/L, median (range)	112.5 (86–153)	112.0 (87–151)	0.673
Total bilirubin, μmol/l, median (range)	84.9 (10.1–440)	79.4 (19.9–423)	0.989
Direct bilirubin, μmol/l, median (range)	64.1 (6.3–347)	69.8 (12.8–367)	0.733
Carbohydrate antigen 19–9, U/ml, median (range)	511.5 (2.0–6021)	493.4 (2.1–5966)	0.834
Tumor size, cm, median (range)	3.5 (2.0–5.0)	3.3 (2.0–5.0)	0.557
Tumor differentiation, case (%)			0.903
Well	2 (4.8)	2 (4.8)	
Moderate	24 (57.1)	22 (52.4)	
Poor	16 (38.1)	18 (42.9)	
N stage, case (%)			0.728
N0	4 (9.5)	5 (11.9)	
N1	38 (90.5)	37 (88.1)	
Adjuvant therapy			0.977
None	4 (9.5)	5 (11.9)	
Gemcitabine	14 (33.3)	12 (28.6)	
Gemcitabine+ Capecitabine	5 (11.9)	6 (14.3)	
S1	12 (28.6)	11 (26.2)	
FOLFIRINOX	7 (16.7)	8 (19.0)	

ASA, American Society of Anesthesiology; BMI, body mass index; FOLFIRINOX, leucovorin and fluorouracil plus irinotecan and oxaliplatin

### Surgical modality

**Fig. 1:**
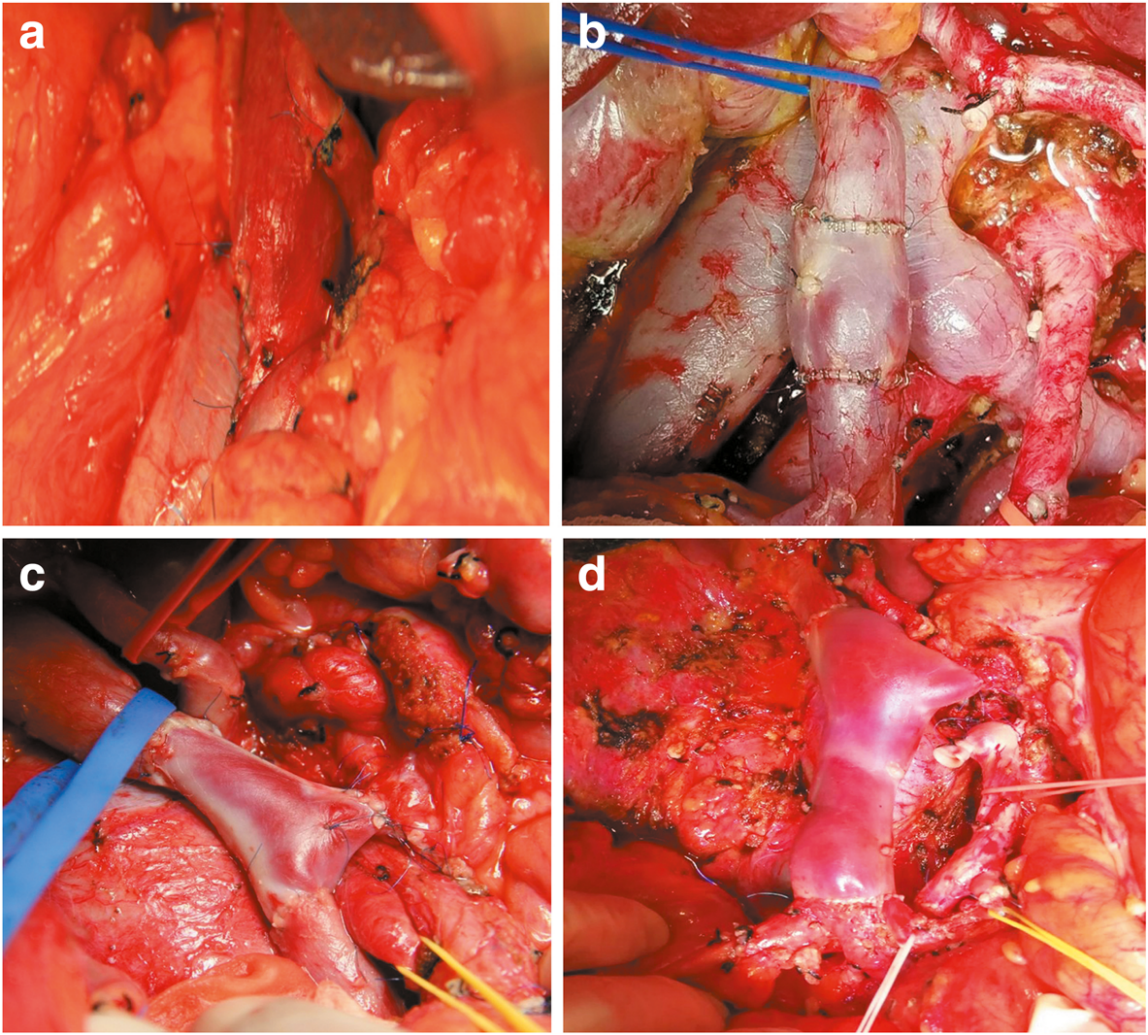
Four types of PV/SMV invasion for pancreatic head cancer. Different reconstruction modality for different type of vascular invasion was presented. **a** Tumor contacted with the PV/SMV of ≤90°, and partial venous excision with direct closure was performed; **b**-**d** Segmental PV/SMV (including superior mesenteric-portal-splenic vein confluence in figure c, and both superior mesenteric-portal-splenic vein confluence and partial first jejunal branch in figure d) resection with reconstruction using allogeneic vein was performed: **b** Tumor contacted with the PV/SMV of > 90° and the length of invasion was > 2 cm. **c** Superior mesenteric-portal-splenic vein confluence was involved; **d** Both of superior mesenteric-portal-splenic vein confluence and branches of superior mesenteric vein (first jejunal branch) were involved

In our center, four types of venous invasion were concluded. Type one, solid tumor contact with the PV/SMV of ≤90°; type two, solid tumor contact with the PV/SMV of > 90°, or obvious stenosis or occlusion of PV/SMV, without extension to confluence of splenic vein; type three, involvement of superior mesenteric-portal-splenic vein confluence; type four, involvement of both of superior mesenteric-portal-splenic vein confluence and branches of superior mesenteric vein. Patients with type one of PV/SMV invasion received partial venous excision with direct closure or partial venous excision using a patch, and patients with type two can received segmental PV/SMV resection with direct end-to-end anastomosis (length of invasion ≤2 cm) or reconstruction using allogeneic vein (length of invasion > 2 cm), whereas for patients with type three or type four, only segmental PV/SMV resection with reconstruction using allogeneic vein can be performed (Vascular reconstruction was completed for pancreatic head cancer with different type of venous invasion, as shown in Fig. 1).

According to the guidelines of ISGPS, standard PD combined with excision of PV/SMV could be defined as extended PD, and all patients underwent extended PD in this study; extended lymphadenectomy including the removal of lymph nodes of stations 5, 6, 8a, 8p, 9, 12a, 12p, 12b1, 12b2, 12c, 13a–b, 14a–d, 16a2, 16b1, and 17a–b was applied for all patients.

### Postoperative management

Subcutaneous injection of nodroparin calcium 0.4 mL per day was started on the second day after operation and ended on the seventh days, then 1 month of oral aspirin was recommended. Doppler B-ultrasound was used for observing the venous blood flow on the third and seventh day after operation. Computed tomographic angiography (CTA) was used for justifying the condition of PV/SMV 1 month after operation, and then 6 months postoperatively; meanwhile Doppler was also used for evaluation of PV/SMV blood flow.

### Parameters for analysis

Demographic and clinicopathologic parameters, operative details, perioperative complications and prognosis recorded to our database were used for analyzed. Drain amylase of > 3 times serum amylase after the third postoperative day, as defined by ISGPS [15], was defined as pancreatic fistula. The status of resection margins was classified based on the International Union Against Cancer R classification, as microscopic radical resection (R0), microscopic positive (R1), or gross positive (R2). Margin < 1 mm was defined as positive margin. Postoperative complications were monitored for 30 days after surgery, and perioperative mortality was defined as death within 30 days after surgery or during hospital stay.

### Follow-up and statistical analysis

Physical examination, laboratory tests, and image examinations were used for follow-up, the follow-up interval was 2 months. SPSS 16.0 (IBM, Chicago, Illinois, USA) was used for data analysis. Quantitative variables were expressed as median and range, and comparisons were analyzed with the Student t-test according to data distribution. Categorical variables were presented as number and percentage and were analyzed by chi-square test or Fisher exact test, as appropriate. Kaplan-Meier method was used to analyze the survival of patients, and the curve of survival between groups was analyzed by log-rank test.

## Results

**Fig. 2:**
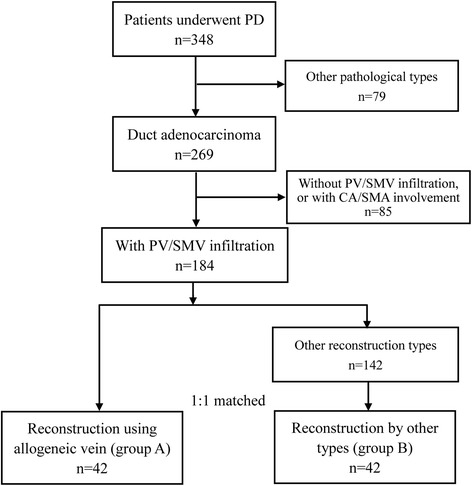
The patients flow in this study

As shown in Fig. 2, from January 2012 to November 2016, a total of 348 patients underwent extended PD in our hospital. Of these patients, 269 cases were confirmed as duct adenocarcinoma of pancreatic head by postoperative pathology. According to the TNM staging of pancreatic cancer of American Joint Committee on Cancer (AJCC), 184 cases had the T3 stage duct adenocarcinoma. 42 received segmental PV/SMV resection with reconstruction using allogeneic vein in all of the 184 patients. Of the 42 patients, type 2 PV/SMV invasion was confirmed in 32 cases, type three in 8 cases, and type four in 2 cases according to our classification of venous invasion (group A).

Patients with reconstruction using allogeneic vein (group A) were 1:1 matched to patients with reconstruction by other methods including segmental excision with direct end-to-end anastomosis, partial venous excision with direct closure, partial venous excision using a patch and segmental excision with artificial vascular graft (group B). In group B, type 1 PV/SMV invasion was confirmed in 17 patients and type two in 25 patients according to our classification of venous invasion. Of the 42 patients, 13 cases received partial venous excision with direct closure, four cases received partial venous excision using a patch, 22 cases received segmental excision with direct end-to-end anastomosis, and three cases received segmental excision with artificial vascular graft. Demographics and clinical characteristics are shown in Table 2. The two groups were well balanced in terms of age, gender, BMI, ASA score, concomitant disease, blood test, postoperative pathology, and adjuvant therapy (Table 1).

**Table 2:** Operation outcomes and long-term outcomes of the two groups

Parameters	Group A (*n* = 42)	Group B (*n* = 42)	*P*-value
Operation time, min, median (range)	490.0 (360.0–920.0)	470 (280.0–650.0)	0.067
Time to vein reconstruction, min, median (range)	52.5 (40.0–80.0)	45.0 (30.0–70.0)	< 0.001
Intraoperative blood loss, ml, median (range)	650.0 (350.0–1650.0)	450.0 (250.0–1350.0)	0.108
Lymph node retrieved	18.5 (7.0–50.0)	18.0 (6.0–46.0)	0.278
Length of vein resected, cm, median (range)	3.0 (2.0–6.0)	2.0 (1.0–4.0)	< 0.001
R1 rate of PV/SMV	2 (4.8)	6 (14.3)	0.137
Postoperative complications, case (%)			
Pancreatic fistula	6 (14.3)	8 (19.0)	0.558
Bleed	2 (4.8)	2 (4.8)	1.000
Delayed gastric emptying	5 (11.9)	4 (9.5)	0.724
Portal vein thrombosis	0	1 (2.4)	0.314
OS, %			0.501
1-year OS	62.9	57.0	
2-year OS	31.5	25.6	
3-year OS	0	0	
DFS, %			0.502
1-year DFS	43.9	36.6	
2-year DFS	10.5	7.4	

DFS, disease-free survival rate; OS, overall survival rate

As shown in Table 2, there was no statistically significant difference was found for operation time between the two groups (490 min vs. 470 min, *P* = 0.067), although the time to vein reconstruction in group A was longer compared with that in group B (52.5 min vs. 45 min, *P* < 0.001). The volume of intraoperative blood loss was 650 mL and 450 mL in group A and group B, respectively (*P* = 0.108). The length of PV/SMV resected was measured in this study, and the median length was 3.0 cm in group A which was obviously longer than that in group B. Although no significant difference for the R1 rate of PV/SMV, the R1 rate decreased almost 10% in group A compared with group B (4.8% vs. 14.3%). The incidences of pancreatic fistula were 14.3 and 19.0%, no significant difference was found (*P* = 0.558), the same result could be found for incidence of bleed (4.8% vs. 4.8%). The stump bleeding of gastroduodenal artery was confirmed for all four patients. Emergency laparotomy was carried out for two patients, interventional embolization was used for another two patients, and none died from bleed. No portal vein thrombosis was found by doppler on the third and seventh day after operation in group A, but one patient had portal vein thrombosis conformed by doppler B-ultrasound. There was no patient with infectious complication related to allogeneic vein.

**Fig. 3:**
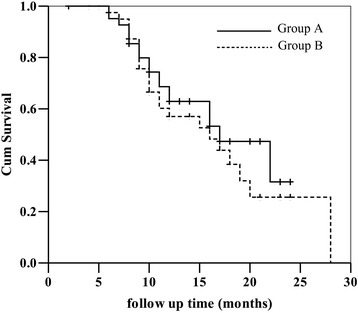
The overall survival rate of the two groups

**Fig. 4:**
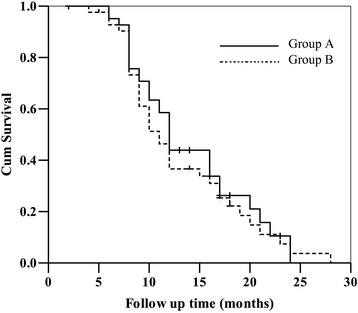
the disease-free survival rate of the two groups

As shown in Fig. 3, the 1- and 2-year OS were 62.9 and 31.5% in group A, and 57.0 and 25.6% in group B (*P* = 0.501). The 1- and 2-year DFS were 43.9 and 10.5% in group A, 36.6 and 7.4% in group B, respectively (*P* = 0.502), which were shown in Fig. 4. Neither group A nor group B had 3-year OS.

## Discussion

Radical resection is still the only potential method for the cure of pancreatic cancer although the development of non-surgical fields [16–18]. There is no unanimous consensus yet on the efficacy of vascular resection for pancreatic cancer with infiltration of PV/SMV [19–21]. Recently, the argument about the value of PV/SMV resection has become more and more prevalent [22]. As an example, a study designed by Murakami Y et al. [12]. Showed that PD with PV/SMV resection and adjuvant chemotherapy in patients with pancreatic head carcinoma might provide good survival without increased mortality and morbidity; whereas a meta-analysis designed by F. Giovinazzo et al. [10]. Revealed that inferior results including increased postoperative mortality, higher rates of non-radical surgery and worse survival were related to PV/SMV resection. Especially to deserve to be mentioned, these two articles were published in the same journal during a short interval. Although the widespread growth of controversy, PV/SMV resection and reconstruction has been still recommended by some centers [9, 22–25].

Four types of venous resection are recommended by ISGPS [14]: partial venous excision with direct closure (venorraphy) by suture closure; partial venous excision using a patch; segmental resection with primary venovenous anastomosis; segmental resection with interposed venous conduit and at least two anastomoses. We think that partial venous excision with direct closure or using a patch may be feasible when venous infiltration is less than 90°, whereas it is not suitable when involvement of PV/SMV is more than 90°. Two main related drawbacks are vessel stenosis and increased R0 rate. We also suggest that segmental resection with direct venovenous anastomosis is preferred when length of invasion ≤2 cm with venous involvement > 90°, direct end-to-end anastomosis may not be the best choice if the length of venous invasion > 2 cm. The disadvantages of end-to-end anastomosis mainly includes increased tension of blood vessel and diameter mismatch. For some infiltration of special location such as superior mesenteric-portal-splenic vein confluence, neither direct end-to-end anastomosis nor artificial blood vessel is suitable. Venous resection and reconstruction by using allogeneic vein may provide an alternative way.

Compared with routine venous resection and reconstruction, the coexistence of advantages and disadvantages can be observed for allogeneic vein reconstruction. We conclude the main advantages of this technique: Firstly, extending surgical indications of pancreatic cancer. Without excessive limitation by infiltration scope of PV/SMV, patients have more chance to receive surgical resection. In our study, we could see that the length of PV/SMV resected in group A was obviously longer than group B. Secondly, improving en-bloc resection rate and R0 rate, more patients can benefit from radical resection. In this study, R1 rate of PV/SMV in group A decreased by nearly 10% compared with that in group B although no significant difference. Thirdly, avoiding incidences of vascular tension after anastomosis, vascular stenosis, anastomotic stricture and diameter mismatch. Recovering normal anatomic structure of vascular for special location such as superior mesenteric-portal-splenic vein confluence to the most extent. Fourthly, avoiding rejection induced by artificial vascular and long-time anticoagulation treatment.

The main disadvantages of this technique are easily observed. Obviously, the procedure of allogeneic vein reconstruction is more complex compared with routine types, thus more operation time is needed. In this study, we could see that the time to vein reconstruction was significantly longer in group A than that in group B, whereas the total operation time and intraoperative blood loss between the two groups were comparable. Preservation of blood vessel is another issue of concern, there has been no unified standard till now.

The complications did not increase in patients underwent allogeneic vein reconstruction, and no increased vein-related complications were found; meanwhile, the prognosis in these patients had slight improvement although no significant difference.

## Conclusions

PV/SMV resection and reconstruction by using allogeneic vein is safety and feasible, it may extend surgical indications and increase R0 resection rate compared with routine types.

## Abbreviations

ASA: American Society of Anesthesiology
BMI: body mass index
CTA: Computed tomographic angiography
DFS: Disease-free survival rate
ISGPS: International Study Group of Pancreatic Surgery
OS: Overall survival rate
PD: Pancreatoduodenectomy
PV: Portal vein
SMV: Superior mesenteric vein
